# Hypofractionated carbon ion therapy delivered with scanned ion beams for patients with hepatocellular carcinoma – feasibility and clinical response

**DOI:** 10.1186/1748-717X-8-59

**Published:** 2013-03-13

**Authors:** Daniel Habermehl, Jürgen Debus, Tom Ganten, Maria-Katharina Ganten, Julia Bauer, Ingo C Brecht, Stephan Brons, Thomas Haberer, Martin Haertig, Oliver Jäkel, Katia Parodi, Thomas Welzel, Stephanie E Combs

**Affiliations:** 1 Department of Radiation Oncology, University Hospital of Heidelberg, INF 400, Heidelberg 69120, Germany; 2 Department of Internal Medicine, University Hospital of Heidelberg, INF 410, Heidelberg 69120, Germany; 3 Department of Diagnostic Radiology, German Cancer Research Center, INF 280, Heidelberg 69120, Germany; 4 Heidelberg Ion Beam Therapy Center (HIT), INF 450, Heidelberg 69120, Germany

## Abstract

**Purpose:**

Photon-based radiation therapy does currently not play a major role as local ablative treatment for hepatocellular carcinoma (HCC). Carbon ions offer distinct physical and biological advantages. Due to their inverted dose profile and the high local dose deposition within the Bragg peak, precise dose application and sparing of normal tissue is possible. Furthermore, carbon ions have an increased relative biological effectiveness (RBE) compared to photons.

**Methods and materials:**

A total of six patients with one or more HCC-lesions were treated with carbon ions delivered by the raster-scanning technique according to our clinical trial protocol. Diagnosis of HCC was confirmed by histology or two different imaging modalities (CT and MRI) according to the AASLD-guidelines. Applied fractionation scheme was 4 × 10 Gy(RBE). Correct dose application was controlled by in-vivo PET measurement of β + −activity in the irradiated tissue shortly after treatment.

**Results:**

Patients were observed for a median time period of 11.0 months (range, 3.4 – 12.7 months). Imaging studies showed a partial response in 4/7 lesions and a stable disease in 3/7 lesions in follow-up CT- and MRI scans. Local control was 100%. One patient with multifocal intrahepatic disease underwent liver transplantation 3 months after carbon ion therapy. During radiotherapy and the follow-up period no severe adverse events have occurred.

**Conclusions:**

We report the first clinical results of patients with HCC undergoing carbon ion therapy using the rasterscanning technique at our institution. All patients are locally controlled and experienced no higher toxicities in a short follow-up period. Further patients will be included in our prospective Phase-I clinical trial PROMETHEUS-01 (NCT01167374).

## Introduction

Photon-based radiation therapy does currently not play a major role as local ablative treatment for hepatocellular carcinoma (HCC). Usually the therapeutic standard is surgical resection or if not possible local ablative treatments such as transcatheter arterial chemoembolization (TACE) and radio-frequency-ablation (RFA). Despite technical improvements in these fields over the last few years there are still patients not suitable for one of these treatments, e.g. because of a liver cirrhosis with decreased liver function due to locally advanced tumors. If none of these therapeutic procedures can be applied or when patients have metastasized disease, standard of care therapy consists of a systemic treatment with the multi-kinase inhibitor sorafenib (Nexavar^®^) which leads to an improvement in overall survival of two months in locally advanced HCC [1].

Photon-based external-beam radiotherapy has been shown to lead to good local control rates in small patient cohorts but its use is mainly based on an individual decision making and its application should currently be designed in clinical trials. One of the most limiting factors is the relatively low radiation tolerance of normal liver tissue, thus leading to clinical evaluation of high precision radiation techniques such as image guidance (IGRT, image-guided radiotherapy) or even particle beam therapy (PBT), including protons (H1) or carbon ions (C12) [2]. High doses are required for long-term control of HCC- lesions but are difficult to apply because of the limited radiation tolerance of the normal liver tissue, even with modern EBRT (External Beam Radiation Therapy) techniques [3]. Therefore carbon ions offer distinct physical and biological advantages. Due to their inverted dose profile and the high local dose deposition within the Bragg peak, precise dose application and sparing of normal tissue is possible. Furthermore, carbon ions have an increased relative biological effectiveness (RBE) compared to photons [4]. A phase-I clinical trial evaluating toxicity and therapy outcome for HCC-patients treated with carbon ions was initiated at our institution. Patients presented in this manuscript were treated according to the study protocol and received a total dose of 4 × 10 Gy(RBE) using carbon ions at our institution which represent the first dose level according to the dose escalation study concept.

## Patients and methods

### Patient selection

Patients were selected according to the clinical trial protocol; main inclusion criteria were histologically confirmed HCC or diagnosis of HCC according to AASLD-guidelines [5] macroscopic tumor, liver-confined disease without extrahepatic disease as diagnosed by CT, MRI, ultrasound and bone scan, minimal distance of tumor edge to the intestines of 1 cm, age ≥ 18 years old and Karnofsky Performance Score ≥ 60. All patients are seen and evaluated in an interdisciplinary setting including specialists from Gastroentereology, Surgery, Radiology and Radiation Oncology.

A total of six patients (n = 6) with one or more HCC-lesions (n = 7) were identified and selected for treatment with carbon ions delivered by the raster-scanning technique at our institution according to our clinical trial protocol. Diagnosis of HCC was confirmed by histology or two different imaging modalities (CT and MRI) according to the AASLD-guidelines. Applied fractionation scheme was 4 × 10 Gy(RBE). No interruptions > 4 days during study treatment were allowed. Median duration of treatment was 9.5 days (range, 8–11 days).

### Patient immobilization

**Figure 1:**
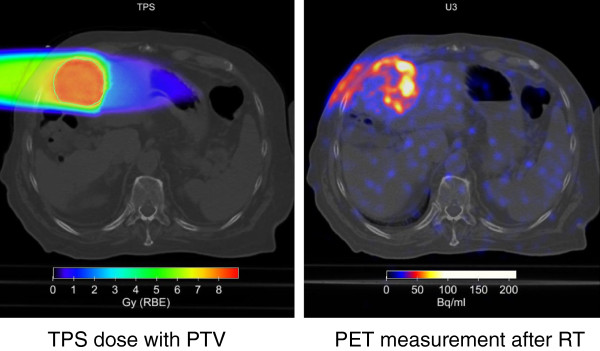
**Measurement of β**^**+**^**-activity at a dedicated PET/CT scanner after application of a dose of 10 Gy(RBE).** Calculation of dose distribution with Syngo^®^ RT planning (TPS).

Patients were immobilized as described previously [6]. In brief, patients were immobilized using an individually shaped vacuum pillow and -in most cases- an abdominal compression to reduce the liver movement. A CT scan was done for treatment planning and the effectiveness of the abdominal compression was measured. On the treatment day patients were repositioned in the above mentioned setting using pen marks and another control CT scan. If the repositioning was adequate the patients were carried to the particle-beam treatment room. One patient received irradiation using respiratory gating. Daily patient positioning was controlled by the comparison of treatment planning *Digitally Reconstructed Radiographs* (DRRs) with kilovoltage images. Correct dose application was controlled by in-vivo PET measurement of β + −activity in the irradiated tissue shortly after the treatment session correlated with CT scans or in selected cases with 4D-CT-scans to account for 4D-dose distribution (Figure 1).

### Treatment planning

For the first patients undergoing hypofractionated carbon ion therapy we decided to use the best known and established immobilization setup since respiratory gating is not yet established at or institution. Furthermore organ motion has to be concerned and leads to dose inhomogenities especially in intensity-modulated radiotherapy and also in particle radiotherapy using the rasterscanning technique. Therefore we aimed to minimize organ and tumor motion to reduce possible interplay effects and dose inhomogenities. In one patient we decided to implement respiratory gating. In this patient a 4D-CT was acquired as previously described. The different single reconstructed standardized respiration phases (at least 8 for one breathing cycle: 0%/40%/70%/100% exspiration and 20%/25%/50%/75% inspiration phases) were analyzed and a time window was chosen where organ motion was relatively low, e.g. period between 70% inspiration and 20% exspiration. We were carefully in implementing this new technique in the experimental setup and have therefore chosen to define a final ITV (internal target volume) that includes the tumour during the whole respiratory cycle and not only the tumor in the above mentioned respiratory phase (period between 70% inspiration and 20% exspiration) with the idea to cover the tumour irrespective of any incertainties during the irradiation procedure. Finally we analyzed the patients’ respiratory curve each treatment day and decided flexibly the time window for irradiation. Live representation of the breathing cycles were monitored with a commercial system using a pressure sensor attached to the waist belt.

Organs at risk such as the intestines, stomach, lungs, kidney, spleen and spinal cord were contoured. Dose constraints of normal tissue were respected according to Pan et al. [7].

The Gross Tumor Volume (GTV) was defined as the area of solid macroscopic tumor contrast enhancement on CT and/or MR-imaging. The Clinical Target Volume (CTV) was defined as the GTV plus a margin of 5 mm. The Planning Target Volume (PTV) included the CTV plus a margin of about 1 cm to account for residual organ motion (all patients underwent contrast enhanced 4D-CT for treatment planning) and setup inaccuracies. C12-RT (carbon ion-radiotherapy) planning was performed using the treatment planning software “Syngo PT Planning” developed by Siemens Oncology Care Systems (OCS, Erlangen, Germany) including biologic plan optimization and was based on the Local Effect Model (LEM) developed by GSI (Gesellschaft für Schwerionenforschung); it is designed for RBE-calculation in different tissue types and for selected endpoints [8]. The optimization of the beam control parameters for the raster scanning technique of the treatment planning system is exerted with respect to the biological effective dose. Treatment was done using a fixed right lateral horizontal beam for all patients in the described time period.

Dose inhomogeneity is a serious topic in scanned beam particle therapy of moving organs. Our attempts to minimize the possible dose inhomogeneities were the use of an abdominal compression to reduce organ and thus tumor motion, the implementation of respiratory gating in one patient, the verification of each treatment plan before definitive treatment and post-treatment 4D dose reconstruction (nota bene in addition to the auto activation imaging). Nevertheless, a recent analysis of the described patients of Richter D. and collegues from our institution (‘4D treatment dose reconstruction for scanned ion beam therapy’, abstract submitted to ASTRO Annual Meeting 2013) revealed a considerable impact of interplay effects on single fraction CTV dose. However, the observed inhomogeneities were clearly reduced for the total applied dose due to the fractionated treatment.

### Follow-up

Patients are currently undergoing follow-up visits with physical examination, laboratory tests and CT-/MRI-scans every four weeks during the first three months and every three months thereafter. Local control (LC) was determined as time period between the first day of radiotherapy and appearance of any local recurrence or progression. Observed toxicity was categorized according to the Common Toxicity Criteria of Adverse Events version 4.03.

### Treatment response

Treatment response was measured and classified as proposed by the AASLD *Panel of Experts in HCC-Design Clinical Trials criteria 2008*[9]:

Complete response (CR): disappearance of any intra-tumor arterial enhancement in all target lesions.

Partial response (PR): at least a 30% decrease in the sum of diameters of the target lesions.

Progressive disease (PD): increase of at least 20% increase in the sum of diameters of target lesions referred to the smallest sum of the diameters of viable target lesions recorded since start of treatment; or appearance of one or more new lesions as per RECIST.

Stable disease (SD) any cases that do not qualify for either PR or PD.

### Ethical approval

The procedures set out in this trial protocol will be performed according to Good Clinical Practice (GCP) and the ethical principles described in the applicable version of the Declaration of Helsinki (2008 Version of the Declaration of Helsinki, adopted at the 59th WMA General Assembly, Seoul, October 2008). The trial will be carried out in keeping with local legal and regulatory requirements. The study plan was submitted to the Institutional Review Board (IRB)/independent Ethics Committee (EC) of the Medical Faculty Heidelberg for approval. Approval of the Bundesamt für Strahlenschutz (BfS) was obtained prior to enrolment into the trial.

## Results

### Local control and survival

Patients were observed for a median follow-up period of 11.0 months (range 3.4–12.7 months). Mean overall survival was 11 months (range 4.0-12.7 months), and only one patient died during follow-up because of a worsening of the underlying liver cirrhosis while the irradiated lesion was stable.

**Figure 2:**
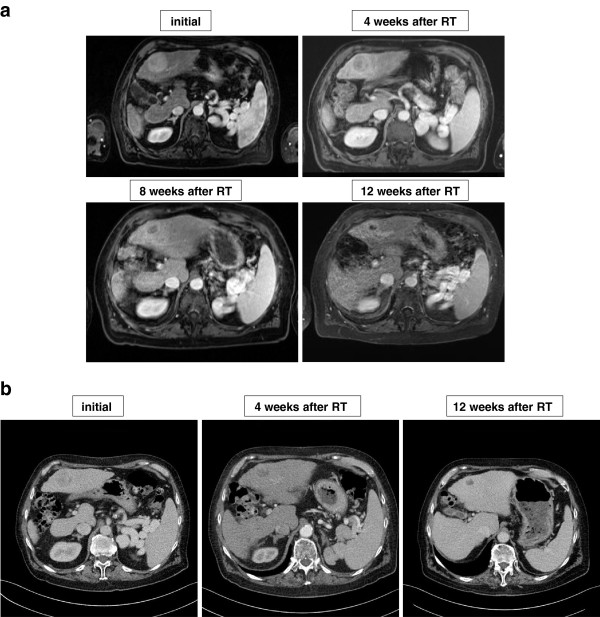
**(a). Treatment response after carbon ion irradiation of a HCC lesion after 4, 8 and 12 weeks (contrast enhanced MRI).** Perifocal contrast enhancement indicates radiation-induced liver reaction. (**b**). Treatment response of the same patient after 4 and 12 weeks (contrast enhanced CT).

Imaging studies based on CT and MRI showed a partial response in 4/7 lesions and a stable disease in 3/7 lesions during follow-up. Median time of local control was 11.3 months (range 2.7-11.9 months) without a local progression of any treated lesion so far (Figure 2a and b). One patient with multiple HCC-lesions had a progression of the non-irradiated lesions after C12-RT and underwent liver transplantation three months after therapy which was initially intended as a bridging therapy (Table 1, patient No. 5). This patient started treatment with Sorafenib (Nexavar^®^) at time of disease progression (of the non-irradiated lesions).

**Table 1:** Detailed patient overview

**Patient no.**	**Gender**	**Age**	**Number of lesions**	**Number of irradiated lesions**	**Max. diameter [cm]**	**Pre-treatment in-loco**	**Histology**	**LC [months]**	**OS [months]**	**Response**	**Comorbidities**	**Comment**
1	F	78	1	1	4.5	none	yes	9.4	10.7	PR	History of breast cancer and stroke, diabetes mell. type II	PROMETHEUS
2	M	63	1	1	2.0	Yes, RFA in loco	no, local relapse, CT + MRI	11.3	11.3	SD	History of metastasized seminoma, AL-amyloidosis type lambda, MALT-lymphoma of the lung, cardiomyopathy, coronary heart disease, diabetes mell. Type II	
3	M	67	1	1	3.3	none	No, CT + MRI	11.8	12.7	PR	diabetes mell. type II, myasthenia gravis, congestive heart failure	
4	F	78	2	2	3.7 and 0.9	none	Yes	11.9	11.9	PR	diabetes mell. type II, COPD II°	
5	M	53	multiple	1	4.0	none	Yes	3.4	3.4	SD	History of renal cell carcinoma, cardiomyopathy, coronary heart disease, diabetes mell. type II	Sorafenib and liver transplantation after RT
6	F	71	2	1	3.4	Yes, PEI and TACE	no, local relapse, CT + MRI	2.7	4.0*	SD	diabetes mell. type II, history of pituitary adenoma, hemochromatosis	Received TACE for 2^nd^ lesion, PROMETHEUS

F = female, M = male, CT = computed tomography, MRI = magnetic resonance imaging, LC = local control, OS = overall survival, PR = partial remission, SD = stable disease, RT = radiotherapy, PEI = percutaneous ethanol instillation, TACE = trans-arterial chemo-embolization, RFA = radiofrequency ablation, * deceased, PROMETHEUS = included in the running clinical trial, COPD = chronic obstructive pulmonary disease.

One patient had two HCC-lesions at time of C12-RT (Table 1, patient No. 6). One of these lesions was heavily pre-treated with repeated PEI (Percutaneous Ethanol Injection) and TACE and the reason for further treatment was a recurrent tumor. The non-irradiated and non-pre-treated lesion was treated four weeks after the end of RT within a first TACE cycle. Child-Pugh-score of this patient was CHILD B and already before initiation of RT hospitalization was needed because of a deterioration of the hepatic encephalopathy (HE). The patient died four months after repeated periods of HE.

The clinical target volume (CTV) of the second patient with a radiological diagnosed recurrence of a previously treated HCC lesion (12 months before) included a suspected residual fibrotic region in which proximity the recurrent tumor was located (Table 1, patient No. 2). This patient had a fulminant intrahepatic progression 10 months after C12-RT with multiple new lesions and newly diagnosed synchronous bone metastases, but the irradiated lesion was controlled.

### Toxicity

Overall tolerance of treatment was well. Five of six patients (83%) reported mild fatigue symptoms. During follow-up none of the patients reported new symptoms. During radiotherapy and the current follow-up period no severe adverse events have occurred. One patient had hepatic encephalopathy and ascites at start of RT and was treated with TACE of a second lesion four weeks after RT, so possible adverse events cannot clearly be assigned to one specific treatment. Subclinical elevations of liver parameters such as ALA, GGT, AP and bilirubin were observed but were almost grade I-II (Table 2). None of the patients had grade-IV or –V toxicities during follow-up.

**Table 2:** Patient and treatment details

**Patient and treatment details**	
**Patients number**	6
**Lesions number**	7
**Gender**	
Male	3
female	3
**Histology**	
yes	4
no	2
**Age [years]**	
Median, range	69 (53-78)
**Child-pugh score**	
**A**	4
**B**	1
**C**	0
**Portal hypertension**	4
**AFP**	
**Positive**	0
**Negative**	6
**Prior treatment of irradiated lesion(s)**	
**Yes**	2
**No**	4
**Tumor size [cm] (median, range)**	3.5 (0.9 – 4.5)
**PTV size [ccm] (median, range)**	243 (40 – 399)
**Toxicity**	
Cholangitis	0
Hematological	
Grade I	1
Grade II	1
Liver enzymes	
Grade I	1
Grade II	1
Fatigue	
Grade I	5
Grade II	0
Grade III	0
**Duration of RT (median, range) [days]**	9.5 (8–11)

RT Radiotherapy, Gy Gray.

## Discussion

Particle therapy delivered as carbon ion therapy using the rasterscanning technique at our institution offers a promising treatment opportunity for patients with both recurrent and primary HCC. Our early results on hypofractionated carbon ion therapy for this indication prove an effective local ablative treatment with a good tolerability. It presents for the first time a radical hypofractionated therapy with local ablative doses using this radiation technique. None of the treated patients experienced a local recurrence during follow-up in addition to a complete absence of any higher grade toxicity (grade IV-V according to CTCAE 4.03). In summary the presented outcome is comparable to previous published series on carbon ion therapy from japanese institutions [2,10].

Results of an early dose-escalation clinical trial from Kato and colleagues were promising with local control rates of more than 90% one year after therapy in 24 patients [10]. The dose-concept consisted of 15 fractions delivered in increasing single doses and starting with an overall dose of 49.5 Gy(RBE). The highest applied dose was 79.5 Gy(RBE). In 71% of all cases tumor response was seen local control rates were 92%, 81% and 81% after 1, 3 and 5 years. No dose-limiting side effects appeared. The authors finally suggest an overall dose of 72 Gy(RBE) to have an optimal gain between high local tumor control and low probability of grade III toxicities.

Until now there is still an apparent lack of prospective studies on particle therapy for HCC: Current treatment recommendations are based on (partially large) retrospective cohort analyses from japanese institutions. A recent study of Komatsu and colleagues reports on 343 patients with 386 tumors treated with proton (242 patients, 278 lesions) or carbon ion therapy (101 patients, 108 lesions) [2]. Carbon ion therapy consisted of 4 different protocols in which 4–20 fractions were applied, with overall doses of 52.8-76 Gy(RBE). Local control rate and overall survival were 93% and 36.3% respectively after a follow-up of 5 years. Multivariate analyses revealed tumor size as the only independent risk factor for a local failure. No higher therapy-related toxicity was observed.

In a recent retrospective analysis Imada et al. analyzed treatment outcome after C12-RT for 18 patients with tumors in proximity of the porta hepatis [11]. This group of patients is of special interest because complication rates are comparably high when standard therapeutic procedures are applied (proximity to large vessels and common hepatic duct). The authors could show that local control after short-course C12-RT of 4 fractions and a total dose of 52.8 Gy(RBE) delivered during 1 week was as high as in the comparison group with tumors not adjacent to the porta (5-year-LC of 87.8% and 95.7% for tumors in the near of the porta hepatis and others, respectively).

Concerning the clinical evidence for the use of particle therapy it has to be remarked that there are currently more significant results derived from proton therapy studies. There are at least three mono-institutional phase-II-studies [12,13]: Patient numbers range from 27 to 75 and the median tumour size ranges from 2.8-5.5 cm. Most patients had underlying liver cirrhosis. The applied hypofractionation protocols are differing and consisted of 15 × 4.2 Gy(RBE) (total 63 Gy(RBE) ) 20 × 3.8 Gy(RBE) (total 76 Gy(RBE) ) [14]. Therapy was overall well tolerated in all three reports and only few grade-III toxicities occurred. Bush et al. report a median PFS of 36 months and a local control rate of 60% after 3 years [12,14]. Eighteen patients benefit from irradiation and underwent liver transplantation during follow-up. In a previous study from the same institution only 1 local relapse occurred after a median follow-up of 31 months and 2-year PFS was as high as 96%.

After C12-RT characteristic morphological changes were observed in the irradiated liver regions and mainly consisted of an increased contrast agent uptake around the target lesions. These phenomena are known from photon-based radiosurgery of liver tumors and are due to well-defined histological changes resembling a focal veno-occlusive disease and its consecutive impact on contrast agent dynamics [15,16]. A detailed analysis of these radiological findings related to C12-RT is in preparation and will be discussed elsewhere.

In comparison to other local ablative therapies such as partial or hemi-hepatectomy or RFA and TACE, outcome of C12-RT does not seem to be inferior to these established treatments and can also be applied in patients with unfavorable factors such as a low performance state, large tumor size, tumor location in proximity to large vessels, severe co-morbidities and a decreased liver function. Nevertheless, the latter factor has to be considered with caution because previous reports point to a possible hepatic impairment after particle therapy, even though no reliable constraints and dose-volume specifications can be defined so far, especially for C12-RT [17]. We observed no severe treatment-related toxicities, especially no clinical relevant signs beside mild fatigue symptoms. In some patients liver enzymes increased with time but it remains unclear if all elevations are uniquely due to C12-RT or to a temporarily decreased hepatic function in the course of the underlying liver disease (liver cirrhosis). Beside, it has to be highlighted that in treated cirrhotic patients the underlying cirrhosis was certainly alcohol-induced whereas cirrhotic Asian patients with the same diagnosis rather have a history of hepatitis.

Our study confirms for the first time the impressive results from our Japanese colleagues. Furthermore the rasterscanning method of beam delivery offers advantages compared to the passive beam delivery technique at the centers in Japan. The delivery of passive beams requires patient-specific beam-modifying devices to achieve an adequate dose distribution to the target structures, which is theoretically associated with a prolonged setup and treatment time. Moreover, conformity of the dose distribution is not always optimal. On the other hand a scanning beam allows improved target volume coverage and sparing of surrounding (normal) tissue. Despite the obvious advantages of scanned beams, moving targets (i.e. the liver) remain a crucial issue because of an increased risk of target misses and misdosage of the target volume due to intra-fractional motion. Compensatory mechanisms include therefore adapted target margins (4D-CT, ITV) and the new implemented respiratory gating [18]. The latter issue is of high importance for the future treatment of our HCC patients and has still to be optimized in cooperation with our colleagues from GSI (Darmstadt).

A further limitation is the fixed 90° horizontal beam line which only allows radiation fields from the right lateral side. All patients have been treated with a single scanned ion beam and dose distribution was acceptable in all cases. Until our 360° gantry for particle beams is in operation – thus improving our beam angle options – the fixed single beam line will be in use.

For the first time we present the feasibility and efficacy of a hypofractionated carbon ion therapy for primary and recurrent HCC delivered with the rasterscanning technique. The complex workflow including patient immobilization, image-guidance, respiratory gating and active scanning beam delivery were successfully implemented at our institution. All patients are locally controlled and experienced no higher toxicities.

## Consent

Written informed consent was obtained from all patients for publication of this report and any accompanying images.

## Competing interest

The authors declare that they have no competing interests.

## Authors’ contributions

SEC, JD and TG have developed the study concept. SEC and JD wrote the study protocol and obtained ethics approval. SEC, DH, JD, TG and MKG provided clinical patient care. OJ, TH and SB performed treatment planning and beam application for carbon ion radiotherapy. SEC, DH and JD implemented the protocol and oversee collection of the data. TH, JD and OJ provided ion beam treatment at HIT. JB and KP performed all PET analyses and substantially contributed to data interpretation. SB made substantial radiobiological analyses and was involved in choosing parameter settings for ion beam treatment planning. MH was responsible for acquisition and evaluation of 4D-data and respiration-gated therapy. TG, MKG and TW were responsible for diagnostic procedures, medical imaging and patient care. ICB made substantial contributions to the data collection. DH performed data analysis and interpretation and wrote the manuscript. SEC helped to write and finalize the manuscript. All authors read and approved the final manuscript.
